# Sensory nerves enhance triple-negative breast cancer invasion and metastasis via the axon guidance molecule PlexinB3

**DOI:** 10.1038/s41523-022-00485-z

**Published:** 2022-11-04

**Authors:** Thanh T. Le, Samantha L. Payne, Maia N. Buckwald, Lily A. Hayes, Savannah R. Parker, Christopher B. Burge, Madeleine J. Oudin

**Affiliations:** 1 Department of Biomedical Engineering, Tufts University, 200 College Avenue, Medford, MA 02155 USA; 2 Department of Biology, MIT, Cambridge, MA 02139 USA

**Keywords:** Metastasis, Cancer microenvironment

## Abstract

In breast cancer, nerve presence has been correlated with more invasive disease and worse prognosis, yet the mechanisms by which different types of peripheral nerves drive tumor progression remain poorly understood. In this study, we identified sensory nerves as more abundant in human triple-negative breast cancer (TNBC) tumors. Co-injection of sensory neurons isolated from the dorsal root ganglia (DRG) of adult female mice with human TNBC cells in immunocompromised mice increased the number of lung metastases. Direct in vitro co-culture of human TNBC cells with the dorsal root ganglia (DRG) of adult female mice revealed that TNBC cells adhere to sensory neuron fibers leading to an increase in migration speed. Species-specific RNA sequencing revealed that co-culture of TNBC cells with sensory nerves upregulates the expression of genes associated with cell migration and adhesion in cancer cells. We demonstrated that lack of the semaphorin receptor PlexinB3 in cancer cells attenuate their adhesion to and migration on sensory nerves. Together, our results identify a mechanism by which nerves contribute to breast cancer migration and metastasis by inducing a shift in TNBC cell gene expression and support the rationale for disrupting neuron-cancer cell interactions to target metastasis.

## Introduction

The breast tumor microenvironment contains an abundance of cell types that contribute to tumor progression. The presence of peripheral nerves in the local microenvironment of epithelial carcinomas was initially described over 30 years ago^1^, and is associated with more aggressive disease in several cancers such as breast, prostate, colorectal, and lung^2–6^. Breast tumors are highly innervated compared to healthy breast tissue^7–9^. Patients with high grade breast cancer have increased number and thickness of nerve fibers, features that were associated with more lymph node metastasis, increased disease recurrence, and poor prognosis^10^. Nerve infiltration is particularly prevalent in triple-negative breast cancer (TNBC), an aggressive subtype of breast cancer^7^. Further, highly aggressive human TNBC tumors are significantly enriched for genes associated with neurogenesis^11^. Given the poor outcomes in patients with TNBC, the presence of nerves in metastatic TNBC tumors, and the important role of cell migration in driving metastasis, it is important that we increase our understanding of the contribution of peripheral nerves to breast tumor cell migration and metastasis.

The peripheral nervous system (PNS) is comprised of sensory, sympathetic, parasympathetic, and motor neurons. Healthy breast tissue is innervated primarily by sensory nerves and secondarily by sympathetic nerves that originate from the thoracic intercostal nerves T3–T5^12–14^. The cell bodies of these nerves are located within the dorsal root ganglia (DRG) of the spinal cord and extend processes toward the topical area of the breast. Sensory nerves receive and transfer sensory information to the central nervous system (CNS), while sympathetic nerves regulate blood supply and lactation during pregnancy^15^. Several studies have demonstrated that sympathetic nerves can regulate breast tumor progression via the immune system by upregulating macrophage infiltration or altering the expression of immune checkpoint molecules, thereby promoting cancer survival and dissemination^16,17^. However, the role of sensory nerves, a major neuronal population in healthy breast tissue, is not well understood in the context of breast cancer progression.

Few physiologically relevant in vitro models are available to dissect the mechanisms of nerve-cancer crosstalk. First, several studies have relied on the use of neuronal-like cell lines such as PC12 or 50B11S^2,8^, however, these cell lines have clear drawbacks. PC12 cells are derived from a pheochromocytoma of the rat adrenal medulla which originates from the neural crest and predominantly differentiate into sympathetic/dopaminergic neurons with nondefinitive axons^18^. 50B11S cells are derived from rat DRG neurons and can be induced to differentiate into mature sensory neurons but their functionality compared to primary DRG is not well characterized^19^. Second, most studies investigating the effect of nerves on breast cancer cells have been focused on the role of soluble cues released by nerves on tumor cells. Several neurotransmitters, such as met-enkephalin, substance P, bombesin, dopamine, and norepinephrine were shown to increase the migration of MDA-MB-468 TNBC cells^20^. Most studies also relied on trans-well assay or the use of neuronal conditioned media, methods which do not allow for direct contact between the two cell types. However, there is evidence that direct interaction between nerves and cancer plays a significant role in nerve-cancer crosstalk. Cancer cells are known to invade and utilize nerve sheath as a route for metastatic spread, a process called perineural invasion^21,22^. In vitro models demonstrate that prostate cancer cells can migrate along nerve fibers^23,24^. In addition, breast cancer cells and neurons can directly interact when they are in close proximity, as has been shown in the context of breast-to-brain metastases where breast tumor cells can form pseudo-synapse with neurons in the CNS^25^. Thus, there is a need for studies that explore the role and impact of direct tumor cell–nerve interactions within the primary breast tumor.

Here, we identified sensory nerves as the most abundant nerve type in human breast cancer. When co-cultured in direct contact with primary sensory neurons in vitro, breast cancer cells attached to neuronal processes and migrated faster than cancer cells alone or in the presence of conditioned media from neurons. Species-specific RNA-sequencing reveals upregulation of cell migration, adhesion, and proliferation pathways in tumor cells co-cultured with sensory neurons. Specifically, we show that the axon guidance receptor PlexinB3, which is upregulated in tumor cells, mediates sensory neuron-driven cell attachment and migration. Our work identifies a mechanism by which nerves contribute to tumor cell migration and metastasis, opening new ways to disrupt nerve-cancer interactions to target breast cancer metastasis.

## Results

### Sensory nerves are abundant in human TNBC tumors

**Fig. 1:**
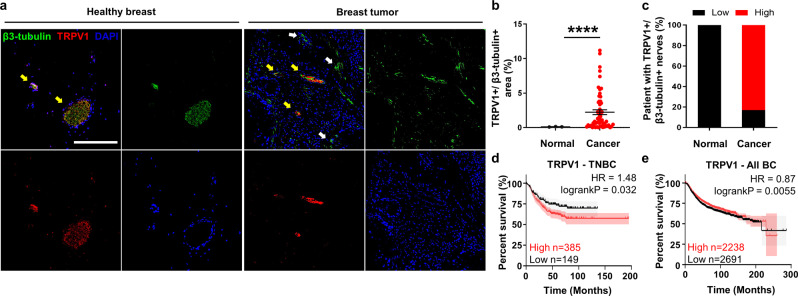
Sensory nerves are abundant in TNBC tumor tissue. **a** Immunostaining of human breast tissue from a tissue microarray stained for β3-tubulin, TRPV1, and nuclei. White arrows show β3-tubulin+ nerve fibers, yellow arrows show sensory nerve fibers positive for both TRPV1 and β3-tubulin (TRPV1+/β3-tubulin+), scale bar denoting 200 μm. **b** Quantification of TRPV1+ and β3-tubulin+ sensory nerve area relative to tumor area (****p < 0.0001, significance was determined by unpaired t test with Welch’s correction). **c** Percent patients with high levels of TRPV1+/β3-tubulin+ sensory nerve presence. **d** Kaplan–Meier curve of TNBC patients comparing outcomes for patients with low or high TRPV1 mRNA expression. **e** Kaplan–Meier curve of all breast cancer patients comparing outcomes for patients with low or high TRPV1 mRNA expression.

We first characterized the presence of sensory and sympathetic nerves in human breast tumor tissue. We performed immunohistochemistry on a microarray of 58 human breast tumor samples from patients with invasive ductal carcinoma. We stained for the pan-neuronal marker β3-tubulin, as well as the transient receptor potential cation channel subfamily V member 1 (TRPV1), which is responsible for nociception in afferent nerves and is highly specific to sensory nerves^26^. In healthy breast tissue, nerves are organized into well-defined sensory nerve bundles, denoted by the colocalization of β3-tubulin and TRPV1 (Fig. 1a, left panels). However, this architecture is drastically changed in breast tumors: nerves are distributed throughout the tissue as individual fibers (Fig. 1a, right panels). There are significantly more sensory nerves (TRPV1+/β3-tubulin+) in breast tumor tissues than in healthy breast (Fig. 1b), with sensory nerves covering on average 2.23% of tumor area. Further, over 80% of patients in our dataset exhibit high level of β3-tubulin+/TRPV1+ signal (Fig. 1c), suggesting that a majority of breast tumors have sensory nerve infiltration.

We then compared the density of sensory nerves to sympathetic nerves, by staining for tyrosine hydroxylase (TH), an enzyme involved in dopamine production and a marker for sympathetic nerves (Supplementary Fig. 1a and Supplementary Fig. 1b). Interestingly, the nerve bundles identified in the healthy breast tissue did not stain for TH, suggesting low levels of sympathetic nerves (Supplementary Fig. 1b). We confirmed there is an increase in sympathetic nerve (TH+/β3-tubulin+) density in breast tumors relative to healthy breast in our cohort (Supplementary Fig. 1c, d), which appeared to have different localization patterns in the tumors than sensory nerves. However, sensory nerves are significantly more abundant in breast tumor tissue compared to sympathetic nerves—2.23% of tissue area coverage for sensory nerves compared to 0.29% for sympathetic nerves (Supplementary Fig. 1e).

We next mined publicly available data sets of TNBC patients to investigate the association of β3-tubulin, TRPV1, and TH with clinical outcome^27^. TRPV1 correlation to outcome has been shown to be cancer-specific: high TRPV1 expression is correlated with more aggressive disease and worse survival in prostate and pancreatic cancer^28,29^, but is associated with improved survival in melanoma^30^. In TNBC, high levels of TRPV1 or TH mRNA were associated with worse outcome in patients (Figs. 1d, S1f). However, there is no association of TRPV1 and TH expression with patient outcome when all breast cancer subtypes are considered (Figs. 1e, S1g). Altogether, these data further motivate investigation into the role sensory nerves play in TNBC.

### Sensory neurons increase TNBC metastasis in vivo

**Fig. 2:**
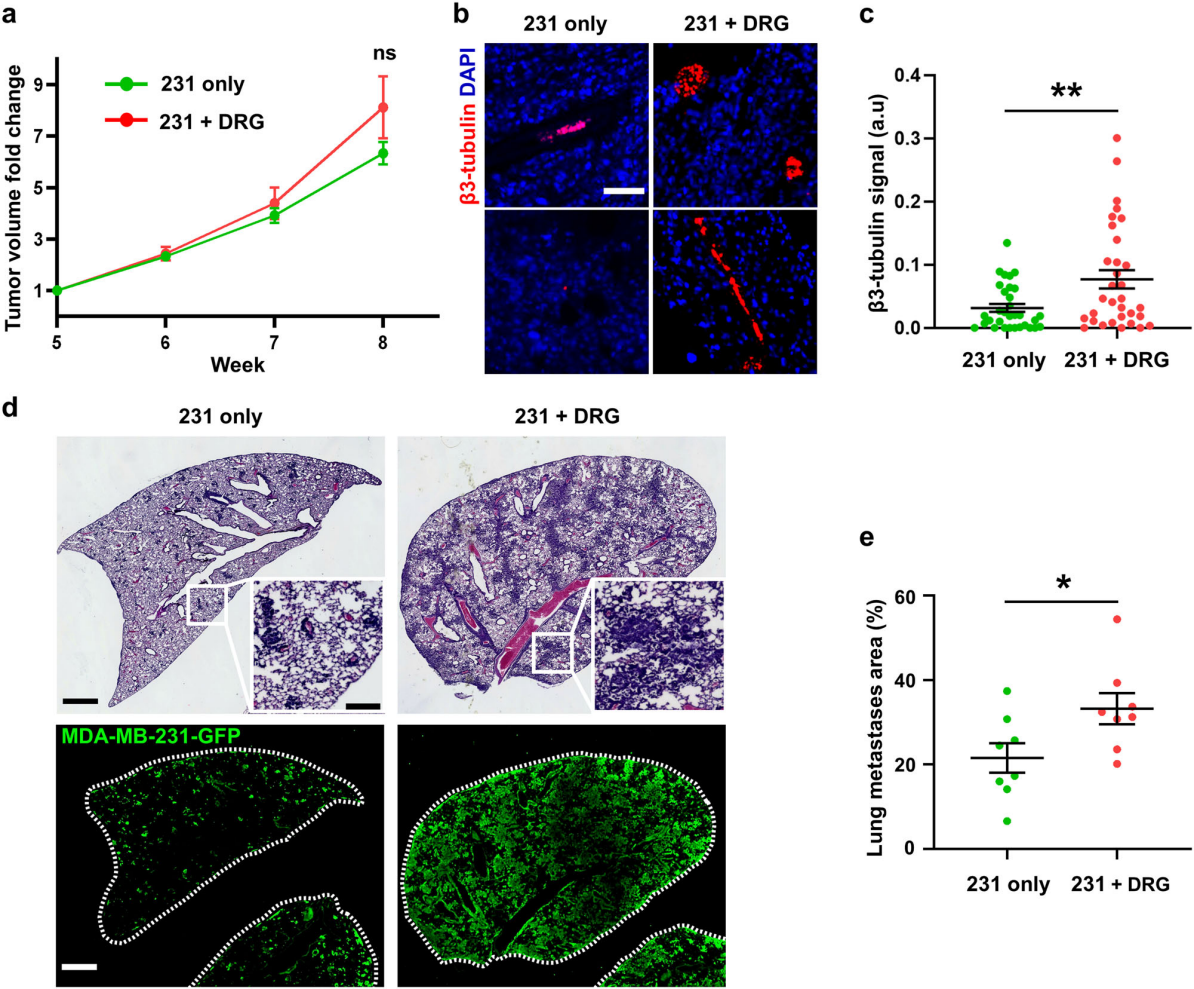
DRG sensory neurons increased metastasis in vivo. **a** Tumor volume of mice injected with GFP tagged MDA-MB-231 cells alone or in combination with DRG sensory neurons measured over time, *n* = 8 for each group. **b** Representative images of immunostaining of mammary tumors for β3-tubulin and nuclei with DAPI, scale bar denoting 50 μm. **c** Quantification of β3-tubulin signal intensity (a.u - arbitrary unit, *n* = 30 field of views across 8 animals). **d** Upper: H&E staining of lung in injected mice, scale bar denoting 1000 μm with insert showing higher magnification view of individual metastases, scale bar denoting 200 μm. Lower: immunostaining of lung metastasis in injected mice stained for anti-GFP antibody, scale bar denoting 1000 μm. **e** Quantification of GFP+ area in lung. Data show mean ± SEM. Significance was determined by unpaired student’s t test (*p = 0.038, **p < 0.01).

To determine if sensory nerves can contribute to breast tumor progression, we investigated the effect of sensory neurons on TNBC tumor growth and metastasis in vivo. Sensory neurons were isolated from the dorsal root ganglia (DRG) of adult female mice using established protocols^31^. We confirmed the identity of the isolated neurons by staining for TRPV1, which is present in the cell body of early stage DRG neurons^32^ (Supplementary Fig. 2a). Murine DRG neurons have previously been shown to survive and innervate tumors when injected subcutaneously with melanoma cells^33^. Here, we injected the mammary fat pad of NOD-SCID-γ mice with GFP-labeled human TNBC cell line MDA-MB-231 (231) alone or 231 cells with isolated DRG sensory neurons. Tumor size of the co-injection group did not significantly deviate from the control group during the experimental period (Fig. 2a). At sacrifice, we showed that there is a significant increase in intratumoral nerve presence in the co-injected group compared to the 231 only group (Fig. 2b, c), suggesting that DRG neurons survived the injection and successfully innervated the primary tumor. Lungs were harvested and stained with H&E and anti-GFP antibody to quantify lung metastasis (Fig. 2d). Injection of DRG neurons together with 231 TNBC cells significantly increased the area of metastases in the lungs (Fig. 2e). These data suggest that the addition of DRG sensory neurons in the primary tumor increase metastasis but not tumor growth in mouse xenograft model.

### Sensory neurons increase TNBC cell migration in vitro

**Fig. 3:**
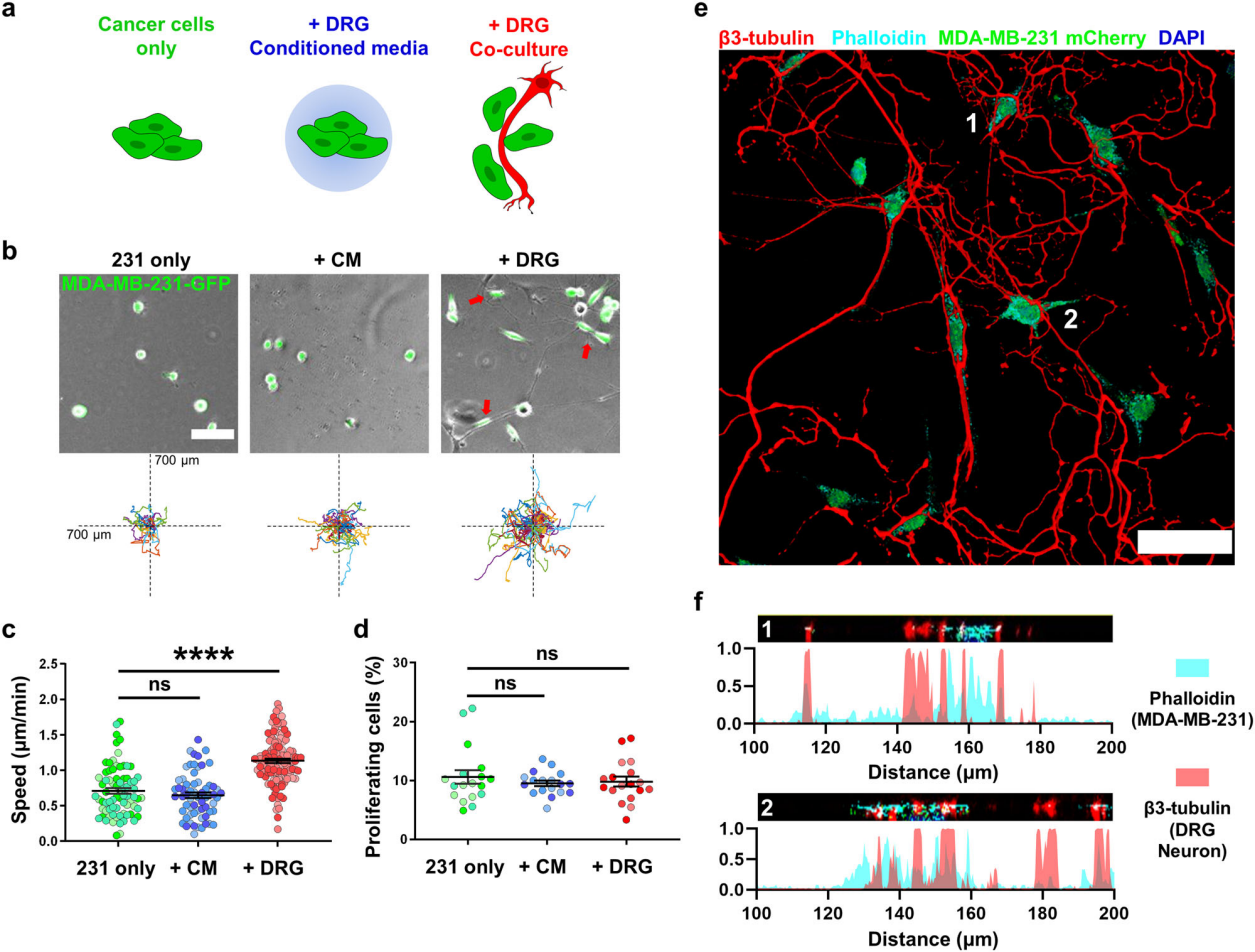
DRG sensory neurons in co-culture increase MDA-MB-231 TNBC cell migration in vitro. **a** Schematic for experimental setup. **b** Representative images and Rose plots displaying the migration track of GFP-tagged 231 cells over 16 h, red arrows denoting cells attached to neuron fibers, scale bar denoting 100 μm. **c** 2D migration speed of 231 cells, each point represents the average speed of one tracked cell over 16h, *n* ≥ 150 cells per condition. **d** Quantification of 231 cells undergoing proliferation in tracking period, each point represents a field of view, *n* ≥ 10 per condition. Data show mean ± SEM. Different shades of color represent cells from different biological replicates. Significance was determined by one-way ANOVA. (**p < 0.01, ****p < 0.0001). **e** Immunostaining of m-Cherry tagged 231 co-cultured with DRG, with β3-tubulin staining for neuron fiber and Phalloidin staining for F-actin. Volume was constructed using 5 μm z-stacks with 0.5 μm step size, scale bar denoting 50 μm. **f** Orthogonal slices of cells numbered 1,2 in **e** (black bar) with plots showing normalized fluorescent signal intensity of Phalloidin and β3-tubulin signal. Distance represents distance across image slice.

To dissect the mechanism by which sensory nerves drive metastasis, we turned to in vitro models. Previous studies have shown that nerves promote tumor phenotypes via secreted factors, but given the close contact seen in our tissue sections between tumor cells and nerves, we wanted to explore the role of direct cell–cell interactions. The proximity of cancer cells to sensory nerve fibers has previously been documented^23,24^, but the impact it could have on tumor cell behaviors is poorly understood. Here, we set up 2 experimental conditions: 1- direct co-culture of DRG neurons with cancer cells and 2- culture of cancer cells with conditioned media collected from 48 h culture of DRG neurons (Fig. 3a). We used 3 cell lines: human TNBC MDA-MB-231 cells stably expressing GFP (231), human TNBC SUM-159 cells stably expressing GFP (SUM159) and mouse PyMT cells isolated from the PyMT-MMTV genetic breast cancer mouse model stably expressing GFP (PyMT) cells. Cell migration is essential for metastasis to occur, contributing to the first step of metastatic cascade to promote movement of primary tumor cells to the vasculature or lymphatics, as well as essential for metastatic colonization^34,35^. We evaluated the effect of nerves or conditioned media on cell migration as well as cell proliferation.

We found that MDA-MB-231 cells cultured directly with DRG neurons at a 1:1 ratio migrate significantly faster and further than breast cancer cells alone and breast cancer cells in conditioned media obtained from DRG neurons (Fig. 3b, c). Conditioned media isolated from the same number of DRG sensory neurons did not elicit a change in the migration speed of MDA-MB-231 cells. Both direct neuron/cancer cell co-culture and conditioned media had no effect on MDA-MB-231 cell proliferation (Fig. 3d). We used confocal imaging to visualize the proximity between cancer cells and the neuron fibers (Fig. 3e), by staining for β3-tubulin which is enriched in neuronal processes, but low in cancer cells and phalloidin which stains the actin cytoskeleton of the tumor cells, which is less abundant in neuronal processes. Line scans through images reveal adjacent signals for both cell types (Fig. 3f), suggesting that TNBC cells come directly into contact with the sensory neurons. In live imaging, TNBC cells utilized the DRG fibers as tracks to move on, spending most of their time (8–16 h) migrating on a neuron (Supplementary Video 1). To determine the effect of direct contact with a neuron in the same breast cancer cells, we tracked cells that migrated on neurons for at least 5 h but then moved off (Supplementary Fig. 2b). Cells on a neuron moved significantly faster than cells off neuron (Supplementary Fig. 2c). For the same cell, moving off the neuron led to a marked decrease in migration speed (Supplementary Fig. 2d). To determine if higher concentrations of soluble factors would have an effect on breast cancer cell migration, we collected conditioned media from DRG sensory neurons seeded at double or quadruple the initial cell density and ratio with cancer cells (20,000 and 40,000 DRG neurons, respectively). When cultured with conditioned media from higher density DRG neurons, TNBC cells migrated faster compared to both the control and the initial condition (Supplementary Fig. 2e, f). However, the magnitude of change is still less than in TNBC cells co-cultured with DRG sensory neurons (1.25- and 1.28-time migration speed fold change in 20,000 and 40,000 DRG cells, respectively, compared to 1.60-time fold change in DRG co-culture).

We then investigated whether the effect of sensory nerves on tumor cell migration was consistent across other TNBC cell line. DRG neurons in direct co-culture also significantly increased cell migration in a second human TNBC cell line SUM-159, with DRG-conditioned media having no effect (Supplementary Fig. 3a–c). To ensure that the observed effects were not due to species differences between the cell lines, we also evaluated the effect of DRG neurons on a murine TNBC cell line derived from the mammary tumors of the spontaneous genetic MMTV-PyMT mouse model. DRG neurons significantly increased PyMT cell migration and proliferation, with DRG-conditioned media having no effect (Supplementary Fig. 3d–f). Together, these data show that TNBC cells move faster upon direct cell–cell contact with sensory neurons.

### Sensory neurons significantly change the transcriptomic profile of TNBC cells and drive the expression of genes associated with cell adhesion and cell migration

Our data show that DRG neurons increase TNBC migration in vitro and metastasis in vivo, but the mechanism by which this occurs remains unknown. The effects we saw require direct cell–cell interaction and were not driven by soluble cues found in conditioned media. Because tumor cells spend several hours at a time in direct contact with neurons, we decided to investigate whether DRG neurons impact gene expression in cancer cells. We performed bulk RNA sequencing of MDA-MB-231 cells alone, cultured with conditioned media from DRG sensory neurons or directly co-cultured with mouse DRG sensory neurons and compared gene expression of MDA-MB-231 cells in each condition. Our pipeline enabled characterization of the transcriptomic profile of human cancer cells and mouse sensory neurons while they are in co-culture, without any trypsinization or sorting steps which could alter gene expression.

We used 2 published species-specific alignment algorithms (S^3^ and Sargasso) to separate reads from mouse sensory neurons from those derived from human TNBC cells^36,37^. Species-specific RNA-seq algorithms work by aligning the bulk RNA-seq of cells from 2 or more different species to their individual reference genomes. These 2 alignments are then compared and any reads that match both genomes, or have low quality score (high mismatch) are excluded. The remaining reads uniquely match the genome of each cell type in co-culture. Previous validation studies have shown that more than 99% of reads are assigned to their correct species^36^. To ensure the accuracy of these algorithms, we mixed breast cancer cell only (231-only) and DRG sensory neurons only (DRG-only) reads in silico to simulate co-culture and ran all three groups individually through the pipeline. The expression profile of TNBC cells from the 231-only group and from TNBC cells separated from in-silico mixing was not statistically different (Supplementary Fig. 4a). We then analyzed our co-culture dataset using S^3^ and Sargasso, which performed similarly for both 231-only and 231+DRG samples (Supplementary Fig. 4b). Both algorithms shared a significant overlap in the differentially expressed genes identified in 231 cancer cells co-cultured with DRG neurons (Supplementary Fig. 4c). These data demonstrate that our species-specific RNA-seq pipeline is robust and consistent.

**Fig. 4:**
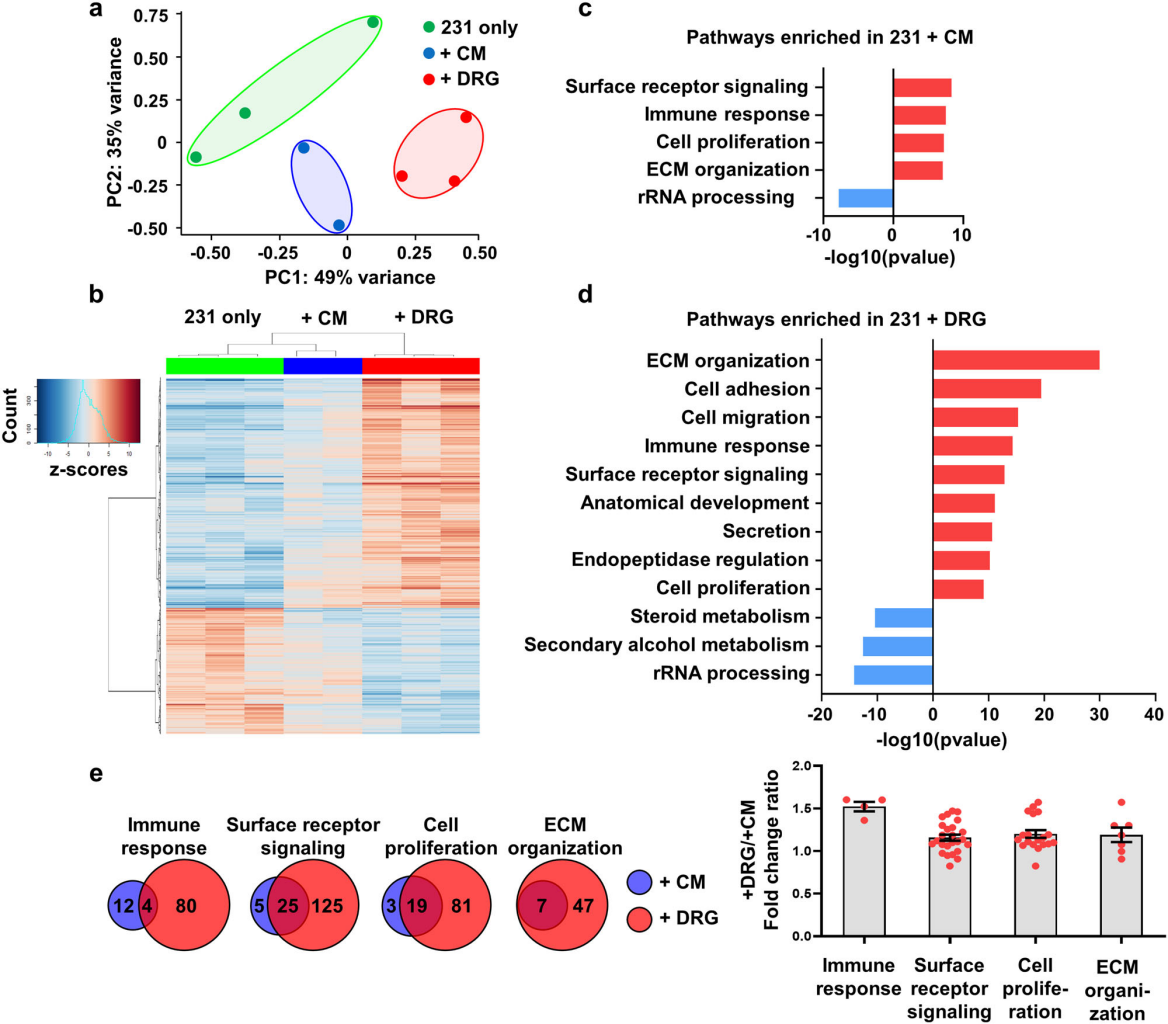
MDA-MB-231 cells undergo a significant change in expression profile when co-cultured with DRG sensory neurons. **a** Principal component analysis of 231 cells gene expression shows distinct clustering among three conditions. **b** Heat map clustering of differentially expressed genes compared to control. **c** Enrichment score for dysregulated pathways in 231 cells cultured with DRG conditioned media. **d** Enrichment score for dysregulated pathways in 231 cells cultured with DRG. **e** Left: Overlap of differentially expressed genes in 231 co-cultured and 231 in conditioned media, with respect to their enriched pathways, right: the ratio of DRG co-culture/conditioned media gene expression fold change for overlapped genes, with each point representing a gene.

Principle component analysis (PCA) shows that MDA-MB-231 cells co-cultured with DRG sensory neurons cluster independently from MDA-MB-231 cells cultured alone or cultured with DRG conditioned media (Fig. 4a). Differential expression analysis indicates distinct gene clusters among 3 conditions, visualized on a heatmap (Fig. 4b) with MDA-MB-231 cells in DRG co-culture having more genes upregulated than downregulated (Supplementary Fig. 4d). Pathway analysis shows that genes associated with surface receptor signaling, immune response, cell proliferation and extracellular matrix organization are enriched in cancer cells cultured in DRG conditioned media (Fig. 4c). Within breast cancer cells directly co-cultured with DRG sensory neurons, the same 4 pathways were also enriched, although at a much higher significance level. In addition, direct cancer cell-DRG culture led to an enrichment in genes associated with cell adhesion and cell migration (Fig. 4d).

For pathways that are enriched in both conditions, we set out to determine whether the same genes were upregulated, but with higher fold change, or whether different genes were involved in the co-culture vs. conditioned media. We found that breast cancer cells in co-culture upregulate 4–8 times more genes within these pathways compared to cancer cells in conditioned media (Fig. 4e). Genes within these pathways that are significantly upregulated by breast cancer cells in both conditions have similar magnitude of upregulation (Fig. 4e). This suggests that cancer cells in direct co-culture with DRG sensory neurons upregulate a distinct set of genes compared to those with conditioned media from DRG neurons. Overall, these data clearly indicate that DRG sensory neurons can induce a substantial change in the transcriptomic profile of TNBC cells, upregulating genes associated with cell migration and adhesion. These data provide the first characterization of the impact of peripheral neurons on TNBC gene expression.

### PlexinB3 regulates sensory nerve-driven TNBC cell migration

**Fig. 5:**
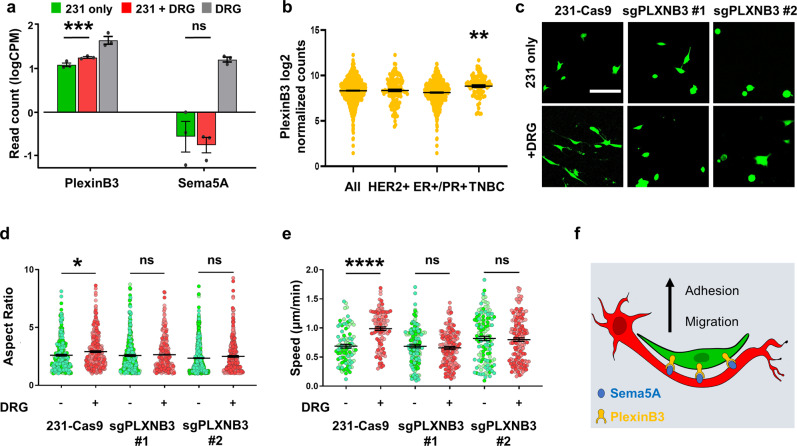
PlexinB3-Sema5A signaling regulates the nerve-cancer crosstalk. **a** Read count of PlexinB3 and Sema5A in 231 cells and neurons (***p < 0.001, significance was determined by EdgeR package). **b** PlexinB3 expression in various breast cancer subtypes, data taken from TCGA. **c** Representative images of 231-Cas9 and 231-sgPLXB3 cells, scale bar denoting 100 μm. **d** Aspect ratio of 231-Cas9 and 231-sgPLXB3 cells, each point represents a cell, *n* ≥ 500 cells per condition. **e** 2D migration speed of 231-Cas9 and 231-sgPLXB3 #1 and #2 cells, each point represents the average speed of one tracked cell, *n* ≥ 150 cells per condition. **f** Schematic for proposed PlexinB3-Sema5A interaction. Different shades of color represent cells from different biological replicates. Data show mean ± SEM. Significance was determined by one-way ANOVA (* = 0.034, **p < 0.01, ****p < 0.0001).

We next set out to identify genes that could be driving the increased migration of 231 cells co-cultured with DRG sensory neurons. Given that the effect is dependent on tumor cell-sensory nerve direct contact, we focused on genes known to be involved in cell migration and adhesion pathways differentially expressed only in TNBC cells in co-culture, but not in conditioned media. The top 10 genes fitting these criteria included several members of the Plexin-Semaphorin family (Supplementary Fig. 4e), genes that are known to be involved in axon guidance in neurons, and have been shown to regulate cell migration in other cell types^38^. PlexinB3 is a receptor whose expression is significantly upregulated in TNBC cells cultured with neurons (Fig. 5a). Plexin B3’s high affinity ligand, Semaphorin 5a (Sema5A), a transmembrane semaphorin is only found to be present in sensory neurons, but is not expressed in cancer cells (Fig. 5a), suggesting that signaling through this receptor would only occur in the presence of neurons^39^. We queried PlexinB3 expression in TNBC patients in the publicly available TCGA database and found that PlexinB3 is specifically upregulated in TNBC patients and not in other breast cancer subtypes (Fig. 5b)^40,41^. Thus, we hypothesized that the tumor Plexin B3-neuron Sema5A interaction mediates sensory nerve-driven breast cancer cell migration.

To investigate this, we knocked down PlexinB3 in MDA-MB-231 cells using CRISPR-Cas9 and 2 separate sgRNAs. Both sgRNAs induced a significant decrease in PlexinB3 mRNA levels compared to the Cas9-expressing control (231-Cas9) (Supplementary Fig. 5a). Knockdown of Plexin B3 in 231 cells did not induce any significant changes in morphology or cell migration speed when tumor cells were cultured alone (Fig. 5d, e). However, while 231-Cas9 cells attached and elongated along DRG neuron fibers, sgPlexinB3 cells remained rounded, quantified by the cell aspect ratio (major axis length/minor axis length) (Fig. 5d). Further, the effect of DRG sensory neurons on cell migration was abrogated in 231-sgPlexinB3 cells (Fig. 5e, Supplementary Fig. 5b, and Supplementary Video 2). Proliferation was not significantly affected by the knock down of PlexinB3 (Supplementary Fig. 5c). Similar results were observed in the human TNBC line SUM159: when PlexinB3 was knocked down (Supplementary Fig. 5d), the cancer cells migrated slower than wild type when co-cultured with DRG (Supplementary Fig. 5e). Taken together, our results identify PlexinB3 as a mediator of DRG sensory nerve-driven tumor cell migration.

## Discussion

In this study, we characterize a mechanism by which direct cell–cell contact between sensory nerves and TNBC tumor cells drives migration. First, we show that sensory nerves are approximately 5 times more abundant than sympathetic nerves in TNBC patient tissue. We show that tumor cells make direct contact with sensory neurons isolated from adult mouse DRGs, which increases their cell migration speed in vitro, and that co-injection of tumor cells with DRGs increases lung metastasis in vivo. We characterized the gene expression profile of cancer cells while in co-culture with sensory neurons by utilizing species-specific sequencing algorithms. We identified a distinct shift in the gene expression profile of cancer cells, with upregulation of cell migration and adhesion pathways. Further, we show that the axon guidance molecule PlexinB3 mediates DRG sensory nerve-driven migration. Overall, these studies provide evidence for the role of sensory nerves in driving tumor progression in TNBC and demonstrate that nerves in the tumor microenvironment can drive significant pro-tumorigenic changes in tumor gene expression.

Our data show that patient breast tumors have increased sensory innervation compared to normal breast tissue, with diffuse nerve infiltration throughout the tissue and that expression of sensory nerve markers is associated with poor outcome in TNBC. The disruption of nerve bundle architecture in breast tumors suggests that cancer cells may stimulate dysregulated outgrowth of existing nerves. Sensory neurogenesis is closely tied to angiogenesis, as both share common pathways and can regulate one another^42^. Breast cancer cells have been shown to release VEGF-A which can induce sensory neuron outgrowth and axonal branching in vitro^8^. Head and neck cancer cells can also release exosomes packed with axon guidance molecules such as EphrinB1 to promote sensory innervation^43^. Alternatively, recent studies have shown that intratumoral nerves can originate from sources other than existing nerves. For example, neuroprogenitor cells from the CNS can cross the blood-brain barrier to infiltrate prostate tumors and differentiate into adrenergic sympathetic neurons^44^. In PyMT-MMTV model of breast cancer, doublecortin positive neural progenitors also infiltrate breast tumors, although their specific identity is not known^44^. Finally, a fraction of cancer stem cells isolated from colorectal and gastric adenocarcinoma cells can be induced to differentiate into parasympathetic and sympathetic neurons which then innervate the primary tumor^45^. Taken together, these studies support further investigation into the origin of the sensory nerves found in TNBC and the mechanisms by which TNBC cells drive an increase in sensory nerve density and neurogenesis.

We also show that DRG sensory neurons drive TNBC cell migration via direct cell–cell contact. Our results suggest that direct contact with nerves significantly increases migration speed, the first step of the metastatic cascade. Cell migration is necessary for dissemination to occur and increasing the migration speed of tumor cells in the primary tumor has been shown to directly correlate with increased metastasis in secondary organs^34,46–48^. Nerves within the primary tumor can directly contribute to increasing local dissemination in the surrounding tissue, leading to increased intravasation and colonization. We found that tumors cells elongate and migrate on DRG sensory neuron processes. Consistent with these results, prostate and pancreatic cancer cells can engage with DRG neurons during perineural invasion (PNI)^21,49^. However, PNI often refers to the infiltration of tumor cells into an existing nerve bundle structure. Here, in breast tumors, we see discrete individual nerves spreading throughout the tumor, suggesting that tumor cells may interact directly with sensory nerves while in the primary tumor before exiting and reaching nerve bundles in the local healthy tissue. Indeed, PNI, as it is defined, is rarely detected and has limited prognostic power in breast cancer^22^. Studying the interactions between single nerves and tumor cells using appropriate in vitro models is critical to understanding the contribution of nerves to local invasion and metastasis.

So far, most studies have shown that neurons promote tumor phenotypes by secreting soluble factors, using assays where neurons are not directly in contact with tumor cells^8,20^. Growth factors such as nerve growth factor and neurotransmitters such as dopamine and norepinephrine which are secreted by neurons have been shown to act as chemoattractants to breast, gastric and pancreatic cancer cells^20,50,51^. However, our in vitro results showed that while co-culture with DRG neurons increased cell migration of both mouse and human breast cancer cell lines, conditioned media from the same density of DRG did not have any observable effects. Interestingly, increasing the concentration of soluble factors by increasing the density of DRG sensory neurons to 2 or 4 times that of cancer cells did lead to increased cancer cell migration. While this result suggests that high concentration of released factors can regulate breast cancer cell migration, it also raises the question of which tumor cell:nerve ratio is physiologically relevant in the breast tissue and in what context do nerves interact with tumor cells in vivo. Based on the immunostaining performed, the density of nerves within tumors is not 2–4 fold higher than the density of tumor cells and breast cancer cells may not see such high concentration of nerve-derived factors in the breast tissue. However, nerves are abundant in tumor rich regions, suggesting that tumor cell-nerve contact does happen in vivo. Our detailed migration analysis shows that nerve-induced changes in cell migration are dependent on cell–cell interactions, and can induce higher migration speeds than the soluble factors. While conditioned media from low density of DRG neurons did not elicit migration changes, it still did significantly affect the gene expression profile of TNBC cells. The differences in genes and pathways upregulated in breast cancer cells under conditioned media and co-culture also suggest that soluble factors have a distinct effect on TNBC cells compared to direct contact. Thus, it is likely that nerve-cancer crosstalk is regulated by both soluble factors and direct cell–cell contact, with our data highlighting that direct nerve-tumor cell contact can elicit higher migration and more extensive changes in gene expression.

Lastly, we show that the axon guidance molecule Plexin B3 is upregulated in TNBC cells in culture with sensory neurons and that it mediates sensory nerve-driven TNBC cell migration. The semaphorin and plexin family is emerging as an important regulator of several cancer phenotypes^52–55^. High expression of Sema5A, the ligand for Plexin B3, is correlated with worse survival in cervical, gastric, and pancreatic cancer^47–49^. However, it is not expressed in our TNBC cells in culture. The mechanisms by which sensory neurons drive changes in tumor cell gene expression remain poorly understood and warrant further investigation. How PlexinB3 expression is induced in TNBC cells is not explored in this study, due to the limited understanding of PlexinB3 signaling pathway in the literature as well as difficulty in differentiating the source of PlexinB3 protein/mRNA with traditional methods in a co-culture model. The downstream mechanisms by which the PlexinB3-Sema5A interaction regulates tumor cell behaviors are also still unclear. PlexinB3 is known to form a complex with and phosphorylate Met when Sema5A is present, promoting migration in HMEC-1 endothelial cells^39,56^. In contrast, PlexinB3 can also inhibit motility by inactivating Rac1 with RhoGDPα^57^. Therefore, while knocking down PlexinB3 inhibited DRG sensory neuron-driven migration, further studies are needed to investigate the mechanisms by which activation of PlexinB3 drives cell adhesion, elongation, and migration.

Sensory nerves have previously been shown to regulate cancer progression: sensory denervation of the pancreas delay cancer onset and progression in an autochthonous mouse model expressing mutant Kras^58^. Co-injection of DRG sensory neurons with B16 melanoma cells accelerate tumor growth in a xenograft model^33^. In in vitro models, proliferation and survival of pancreatic cancer cells are enhanced while in co-culture with DRG sensory nerves^59^. Sensory nerves can also facilitate the migration of prostate cancer cells by providing physical support for cells to move on^23,24^. On the other hand, recent studies have reported the anti-tumor role of sensory nerves. The sensory nervous system is a known immune regulator^60^. In melanoma and breast cancer, activation of sensory nerves through TRPV1 agonist or genetic modulation increased the recruitment of cytotoxic T cells and IL-17 production in the primary tumor^61,62^. All these studies have used different strategies to manipulate sensory nerves, by regulating their presence or activity, and it will be important to further dissect how different properties of sensory nerves contribute to their effects on cancer, whether their presence alone, activity, or electrical properties can be individually modulated to fine tune effects on cancer cells.

There are certain limitations to our current study. Here, we used a 2D in vitro model of tumor cell-nerve co-culture. However, a 3D model would better mimic the local breast environment, incorporating components of the breast extracellular matrix such as Collagen I. Others have reported similar findings where pancreatic cancer cells exhibited movement towards and along DRG nerve fibers in a 3D Matrigel model^51,59^. In addition, while in vitro models have allowed us to explore certain aspects of nerve-cancer interaction, the role of PlexinB3 in nerve-driven metastasis will need to be validated in in vivo models. Intravital live imaging studies would also be important in observing tumor cell-nerve interactions in vivo. Finally, sensory nerves induced a range of significant gene expression changes in breast cancer cells. While we focused on Plexin B3, it will be important to perform further experiments to better understand if and how these other pathways are also involved in mediating tumor cell–nerve interactions.

Overall, we identify a mechanism by which nerves regulate breast cancer progression. Nerves offer potential for new clinical approaches to treat metastatic disease. In human patients, there is evidence that usage of inhibitory neurological drugs such as beta-blockers or tricyclic antidepressants are associated with less metastasis and improved survival^63–66^. A better understanding of nerve-cancer crosstalk is the crucial first step in repurposing existing drugs to target metastatic breast cancer.

## Methods

### Immunohistochemistry

Tumors and lungs of mice were fixed in 4% paraformaldehyde for 24 h, followed by 70% ethanol for 24 h and embedded in paraffin. Sectioning was performed using microtome with 10 μm thickness. Sections were then deparaffinized and Citra Plus solution (HK057, Biogen, Fremont, CA) were used for antigen retrieval. Blocking was performed in PBS with 0.5% Tween-20 and 10% donkey serum. Antibodies were diluted in PBS with 0.5% Tween-20, 1% donkey serum in their respective concentration. Sections were incubated with primary antibodies overnight at 4 °C, followed by fluorophore-conjugated secondary and DAPI for 2 h. For in vitro assays, cells were fixed in 4% paraformaldehyde for 15 min and permeabilized with 0.2% TritionX-100. Blocking was performed in PBS with 5% BSA. Cells were then incubated with primary antibodies overnight at 4 °C followed by flourophore-conjugated secondary and DAPI for 1 h. Imaging was performed using a Keyence BZ-X710 microscope (Keyence, Elmwood Park, NJ) and quantification was done using ImageJ (National Institute of Health, Bethesda, MD). For confocal imaging, Leica CARS SP8 system (Leica, Buffalo Grove, IL) managed by Tufts Advanced Microscopic Imaging Center was used.

Primary antibodies and their concentration used include: 1/800 rabbit anti-beta-3-tubulin (ab18207, Abcam, Cambridge, MA), 1/800 mouse anti-beta-3-tubulin (T5758, Sigma, St. Louis, MO), 1/100 mouse anti-mouse-TRPV1 (sc-398417, Santa Cruz Biotechnology, Dallas, TX), 1/100 rabbit anti-human-TRPV1 (ab3487, Abcam, Cambridge, MA), 1/200 mouse anti-tyrosine-hydroxylase (T1299, Sigma, St.Louis, MO), 1/200 rabbit anti-GFP (A-11122, Thermo Fisher Scientific, Waltham, MA), 1/1000 DAPI (D1306, Thermo Fisher Scientific, Waltham, MA). 1/250 Alexa Flour 633 Phalloidin (A22284, Thermo Fisher Scientific, Waltham, MA).

### Survival analysis

Survival data was obtained from mRNA gene chip data of breast cancer patients from Kaplan–Meier plotter^27^. TNBC status was determined by ER/PR/HER2 IHC status. The cutoff values were calculated using “best cutoff” option^67^. The mRNA expression values for each group are as follow: TRPV1 TNBC: TRPV1 low (3–28), TRPV1 High (29–507); TRPV1 All Breast Cancer: TRPV1 Low (1–45), TRPV1 High (46–1017); TH TNBC: TH Low (1–12), TH High (13–3862); TH All Breast Cancer: TH Low (1–9), TH High (10–3862).

### Cell lines

MDA-MB-231-GFP, SUM159-GFP, and HEK293T cells were obtained from ATCC (Mannassas, VA). 231-GFP and HEK293T cells were cultured in DMEM (MT10013CV) with 10% FBS (SH30071.03, Cytiva, Marlborough, MA) and 1% PSG (10378061). SUM159-GFP cells were cultured in F-12 (11765062) with 5% FBS, 1% PSG, 5 µg/ml insulin (12585014), 1 µg/ml hydrocortisone (H0888, Sigma, St. Louis, MO), 20 ng/ml EGF (PHG0311). PyMT-GFP cells were a gift from Prof. Richard Hynes’s lab at MIT and were cultured in a 1:1 mix of DEM and F-12 with 2% FBS, 1% BSA (A2153, Sigma, St. Louis, MO), 1% PSG, 10 µg/ml insulin, 10 ng/ml EGF. All cell lines were used between p5 and p15 and routinely checked for mycoplasma with Universal Mycoplasma Detection Kit (30–1012 K, ATCC, Mannassas, VA). All media supplements were purchased from Thermo Fisher Scientific, Waltham, MA unless specified otherwise.

### DRG dissection and co-culture with cancer cells

Following published procedure^31^, DRG were dissected from 7 weeks old SW-F female mice (Taconic, Albany, NY). In short, mice were euthanized by CO_2_ and the spinal columns were isolated. Lateral cuts were then made to expose the spinal cord and ganglia that lie adjacent to the spinal cord were collected. Single cell dissociation of the DRG were performed with 1.25 mg/ml collagenase A (10103586001, Sigma, St. Louis, MO) in HBSS followed by 1.2 mg/ml trypsin in HBSS and gentle pipetting. DRG were suspended in neurobasal media which consist of: Neurobasal media (21103-049), 2% B27 (A3582801), 1% GlutaMAX (10569010), 1% Antibiotic-Antimycotic (15240062), 25 ng/ml NGF (450-01, Peprotech, Cranbury, NJ), 10 ng/ml GDNF (PHC705). 24-well plate with glass bottom was coated with 0.1 mg/ml PDL (P0899, Sigma, St. Louis, MO) and 20 μg/ml laminin (L2020, Sigma, St. Louis, MO). 10,000 dissociated cells from DRG were seeded into each well, and neurobasal media was changed every 48 h. Media was collected and filtered with 0.2 µm syringe filter to be used as conditioned media with 1:1 ratio of DRG conditioned:fresh media. To increase the concentration of soluble factors, DRG were seeded with higher density (20,000 and 40,000 cells) and conditioned media was similarly collected and used. 72 h after seeding DRG, cancer cells were trypsinized, counted, and resuspended in neurobasal media. 10,000 cancer cells were then seeded into wells with DRG or empty wells precoated with PDL and laminin as control. All media supplements were purchased from Thermo Fisher Scientific, Waltham, MA unless specified otherwise.

### 2D migration assay

24 h after co-culture was established, cells were put into an optically clear incubating chamber within the Keyence BZ-X710 and imaged every 10 min for 16 h. Tracking the movement of fluorescently labeled cancer cells was done semi-automatically using VW-9000 Video Analysis Software (Keyence, Elmwood Park, NJ). The resulting position data was then compiled, plotted, and calculated for migration speed using a custom MATLAB script (version vR2020a, Mathworks, Natick, MA). From the time-lapse video, mitotic events from cancer cells were also counted and normalized with total number of cells in the field of view to quantify cell proliferation. For tracking the migration of individual cancer cells when they are on/off neuron fibers, the ImageJ package Manual Tracking was used. Only cells that were on neuron fiber then disengaged from fibers for at least 5 h (30 frames) are tracked.

### Animal experiment

All animal procedures were reviewed and approved by the Tufts University Institutional Animal Care and Use Committee. All mice were housed 5 mice per cage in an on-site housing facility with access to standard food, water and 12/12 light cycle. Immunodeficient NOD-SCID-γ female mice (005557, Jackson Laboratory, Bar Harbor, ME) were housed in sterile housing in separate room. 1 × 10^6^ MDA-MB-231-GFP cells and 2 × 10^5^ DRG cells from SW-F mice (dissected and processed in the same day) were suspended in 100 μl solution of 20% collagen I in PBS. 7 weeks old NOD-SCID-γ female mice were anesthetized with isoflurane and injected with cell solution in the right fourth mammary fat pad, with *n* = 8 mice for each group. Tumor size was measured every week by caliper in duplicate. Mice were euthanized by CO_2_ 8 weeks after injection and tumors and lungs were excised. Lungs were perfused with 4% PFA before fixation.

### RNA sequencing and data analysis

RNA from three biological replicates of MDA-MB-231 Control, DRG Control, MDA-MB-231 in DRG co-culture, and 2 biological replicates of MDA-MB-231 in DRG-conditioned media (1 replicate was excluded during analysis after clustering revealed it was an outlier) was extracted 24 h after co-culture using RNA MiniPrep Kit (R1057, Zymo Research, Irvine, CA). Samples were confirmed to have RIN value >8.5 with Agilent Bioanalyzer before undergoing library preparation with TruSeq stranded mRNA kit by Tufts Genomics Core. RNA sequencing was run on NextSeq550 platform at read length of 150 nt, paired-end, with depths ranging from 28 to 35 million reads per mono-culture samples and 55–72 million reads per co-culture samples.

Quality control of raw reads was performed using FastQC^68^, with adapter trimming and read filtering done using Trimmomatic^69^ using standard settings. Reads were then aligned to both the human genome (assembly GRCh38) and mouse genome (assembly GRCm38) using STAR^70^ with default settings. Both mapped files were then used to separate the expression of human and mouse cell lines in co-culture using S3 or Sargasso algorithm with strict filtering settings^36,37^. Final read counts were quantified using featureCounts^71^. Mono-culture samples were also processed using the same pipeline for consistency. Raw reads from MDA-MB-231 and DRG Control were mixed in-silico and processed to compare with reads from Control only samples to validate the accuracy of both species-specific sequencing algorithms. After confirming that both S3 and Sargasso pipelines yielded similar results, further analysis was done using S3 output due to its stricter filtering.

Gene expression analysis was done using EdgeR R package^72^: genes with CPM less than 1 were excluded. Principle component analysis was calculated with prcomp() command using the TPM values of filtered genes. Differential expression analysis was performed with a fold change threshold of 1.2. Heatmap was constructed using differentially expressed genes. Intra-group means and standard deviations were calculated, then averaged to calculate genes z-scores. Pearson correlation was used to calculate distance metric and Ward’s method was used to cluster genes. Pathway analysis was performed using GSEA and Gorilla for biological ontologies^73,74^ and results were summarized using Revigo^75^.

### PlexinB3 knockdown

For PlexinB3 knockdown in 231 cells using CRISPR-Cas9, HEK293T cells were used to create lentivirus carrying FUCas9Cherry plasmid, a gift from Marco Herold (Addgene plasmid #70182; http://n2t.net/addgene:70182; RRID:Addgene_70182)^76^. MDA-MB-231-Cas9 cells were created by transducing wild type MDA-MB-231 cells with this virus in DMEM and 10 µg/ml polybrene, and centrifuge for 1 h at 800 g. Cells were FAC sorted for mCherry positivity at Tufts GSBS. For knocking down PlexinB3, 2 guide RNAs were used. PLXNB3 sgRNA#1:5′-GGTGTTGGACCAAGTCTACA-3′, PLXNB3 sgRNA#2: 5′-CGACGTGACGCCGTACTCCA-3′. Guide RNAs were inserted into plasmid containing puromycin resistance gene and cloned using Stbl3 Competent E.Coli (C737303, Thermo Fisher Scientific, Waltham, MA). Plasmids were sequenced to confirm insertion (primer sequence 5′-TGCAGGGAAAGAATAGTAGAC -3′), and virus production with HEK293T cells and transduction of MDA-MB-231-Cas9 were conducted as described above. Successfully transduced cells were selected by the addition of 0.5 µg/ml puromycin into the media. To achieve uniform and stable PlexinB3 knock-down, clonal expansion was conducted where approximately 1 cell was seeded into an individual well in a 96-well plate, and only wells with single colony are expanded for further use. For PlexinB3 knockdown in SUM159 cells using siRNAs, 3 siRNAs were pooled: 5′-ACAUGAGCCAAGCUGUCAUAG-3′, 5’-UAAAGAGCAUGGGUGUUGUCC-3′, 5′-UGUUGUUGUAGUCGGAGAAGUCGCC-3′. Transfection was performed on 100k SUM159 cells using the pooled siRNAs, lipofectamine (13778030, Thermo Fisher Scientific, Waltham, MA) and OptiMEM media (11058021, Thermo Fisher Scientific, Waltham, MA). Cells were used for downstream assays after 48 h incubation. PlexinB3 knock-down was confirmed using qPCR with PlexinB3 primer: forward 5′-ATCGGACGAAGACTTGACC-3′, reverse 5′-CCATCTGGGACCTTGTAGTG -3′ and control GAPDH primer: forward 5′-CCTGCACCACCAACTGCTTA-3′, reverse 5′-GGCCATCCACAGTCTTCTGAG-3′.

### Morphological analysis

24 h after co-culture was established, cells were fixed with 4%PFA and stained with DAPI. Images were taken at 20× with approximately 30 field of views per condition. Wild type MDA-MB-231 cells (non-mCherry) were used as negative control and exposure was adjusted to limit autoflourescence. CellProfiler v3.1.8^77^ was used to identify cell shape: DAPI was used to identify individual cell then mCherry was used to determine cell shape parameters.

### Statistical analysis

GraphPad Prism v8.4.3 was used for generation of graphs and statistical analysis. To compare between two groups, unpaired two-tailed Student’s t-test was used and a *p*-value of ≤ 0.05 is considered significant. To compare between multiple groups, one-way ANOVA with Tukey’s multiple testing correction was used with a corrected *p*-value of ≤0.05 is considered significant. For RNA-seq, adequately expressed genes passing a fold change threshold of 1.2 and with *p* value ≤ 0.05 in edgeR analysis were considered differentially expressed. Pathways with *p* value ≤ 0.05 and FDR ≤ 0.01 were considered differentially regulated.

### Reporting summary

Further information on research design is available in the Nature Research Reporting Summary linked to this article.

## Supplementary information

Supplementary Information

Reporting Summary

Supplementary Video 1

Supplementary Video 2

## Data Availability

RNA-seq data which include raw sequencing file, aligned, and processed read counts, and differentially expressed genes summary are publicly available in GEO under accession code GSE180508. Other data supporting the findings of this study are available from the corresponding author upon reasonable request.

## Code availability

Custom codes that were used in the analysis of cancer cell migration and RNAseq data have been described above and are available from the corresponding author upon reasonable request. Softwares used and their versions include VW-9000 Video Analysis Software, Matlab vR2020a, FastQC 0.11.9, Trimmomatic 0.32, STAR 2.7.3a, S^3 1.0.1, Sargasso 2.0, EdgeR 3.15, GSEA 4.0.3, Gorilla, Revigo, Graphpad Prism 9, ImageJ 1.53f51.
