# Supplementary material for: Sensory nerves enhance triple-negative breast cancer invasion and metastasis via the axon guidance molecule PlexinB3
Source: NPJ Breast Cancer. 2022 Nov 4;8:116. doi: 10.1038/s41523-022-00485-z (PMC9636220; doi:10.1038/s41523-022-00485-z)
Supplement: Supplementary file 1 — Supplementary Information [file 41523_2022_485_MOESM1_ESM.pdf]

Supplementary Video Legends

Supplementary Video 1. MDA-MB-231 cells migrate faster when in co-cultured with DRG sensory neurons. 231 cells in co-culture elongate along DRG nerve fibers and migrate faster than both 231 control cells and 231 cells in DRG conditioned media.
Supplementary Video 2. PlexinB3 knockdown attenuates the nerve-driven migration of MDA-MB-231 cells. mCherry positive 231 cells are visualized by faux green color. Upper panels: Knocking down PlexinB3 does not have an effect on 231 cells migration when in monoculture, lower panels: knocked down cells migrate slower and have more rounded morphology when in contact with DRG sensory neurons.

Supplementary figures

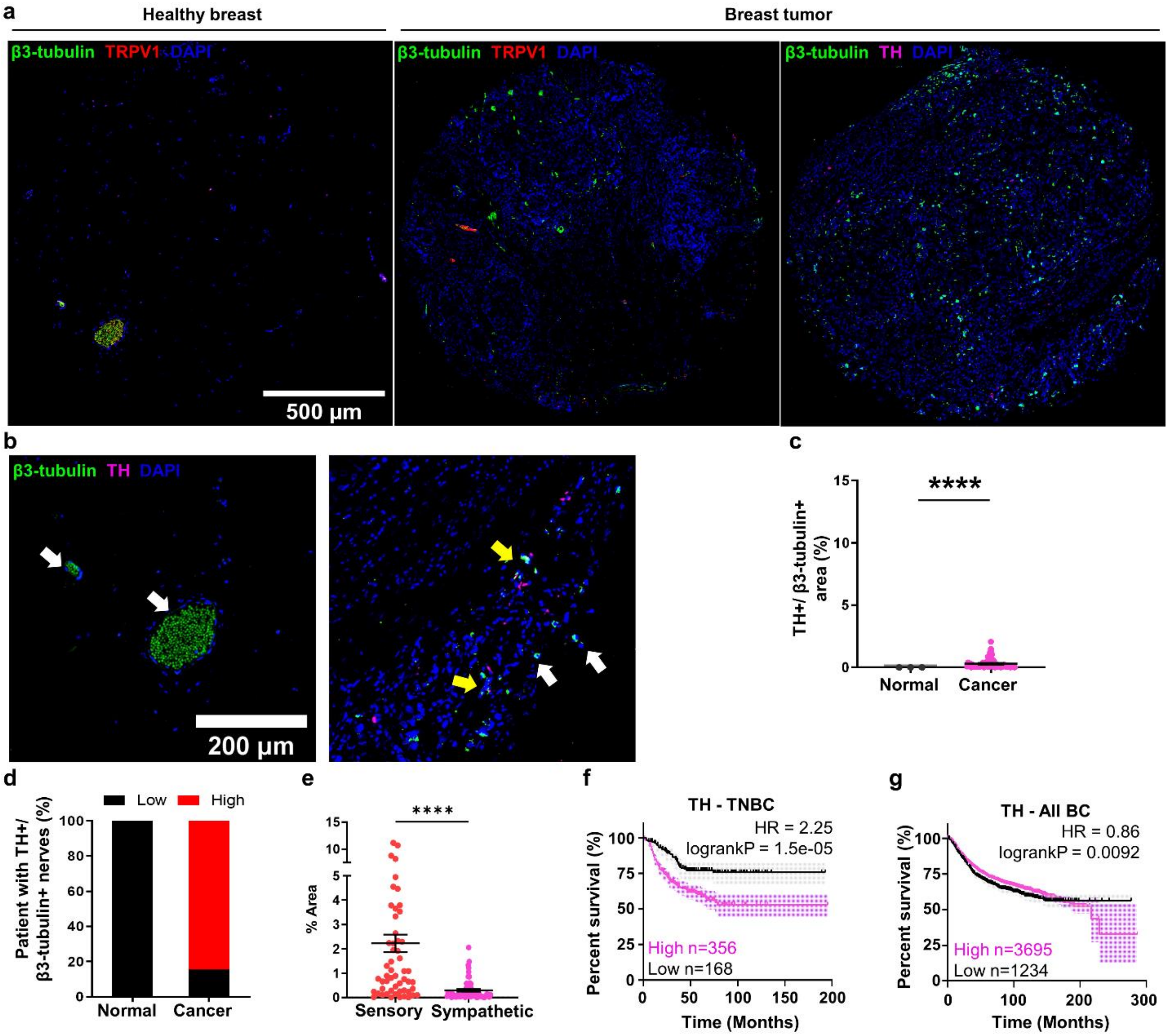

Supplementary Figure 1: Sensory and sympathetic innervation is prevalent in breast cancer patients. a) Immunostaining of human breast tissue from a tissue microarray stained for β3-tubulin, TRPV1, TH and nuclei (macro view). b) Higher magnification images of breast tissues stained for β3-tubulin, TH and nuclei. White arrows denoting β3-tubulin+ nerve fibers, yellow arrows denoting TH+/ β3-tubulin+ sympathetic nerve fibers c) Quantification of TH+/ β3-tubulin+ sympathetic nerve area coverage. d) Percent patients with high levels of TH+/ β3-tubulin+ sympathetic nerve. e) Comparison between the signal area of TRPV1+/ β3-tubulin+ sensory nerve and TH+/ β3-tubulin+ sympathetic nerve in breast tumor tissues. (****$p<0.0001$, significance was determined by unpaired t test with Welch's correction). f) Kaplan-Meier curve of TNBC patients comparing outcomes for patients with low or high TH mRNA expression. g) Kaplan-Meier curve of all breast cancer patients comparing outcomes for patients with low or high TH mRNA expression.

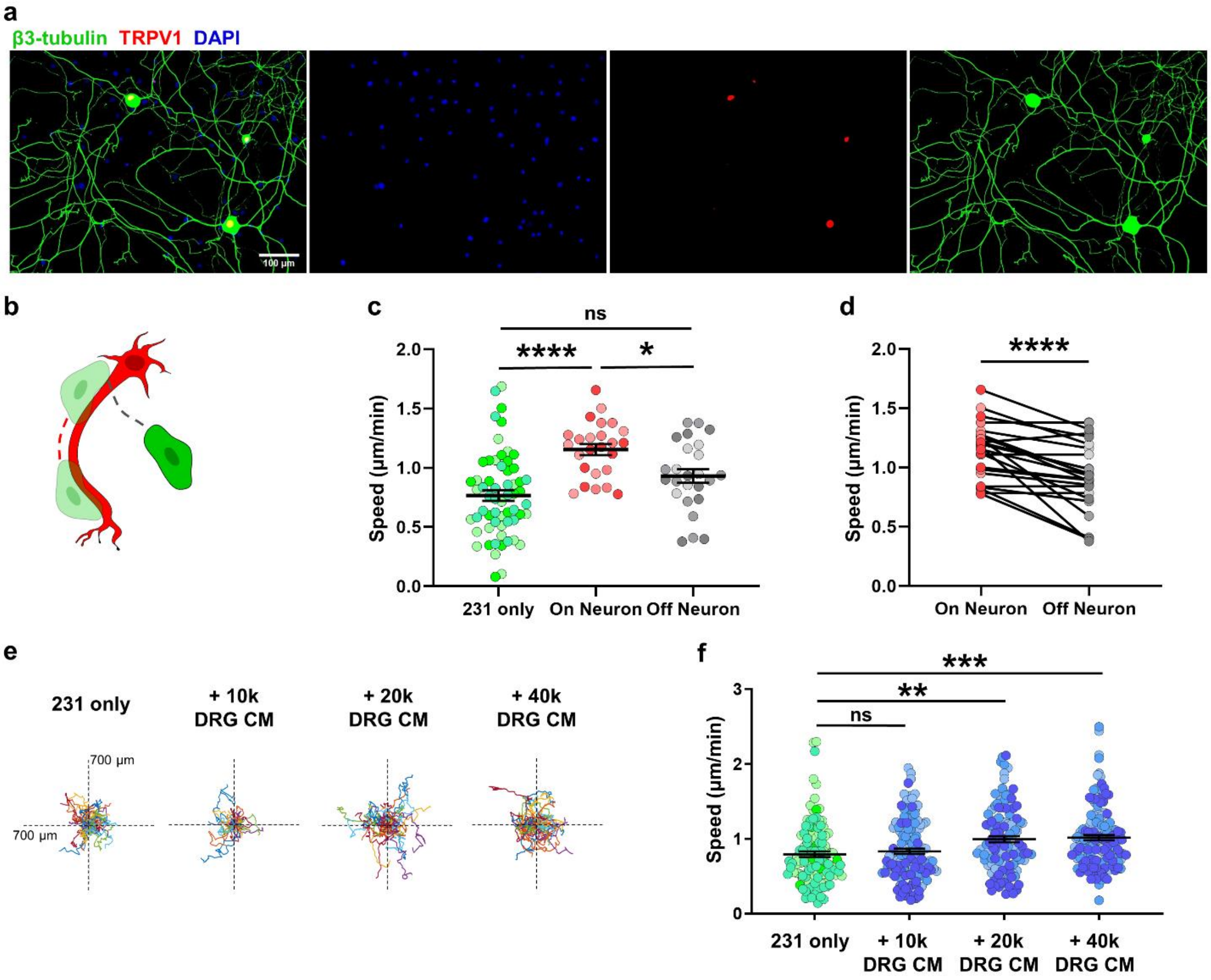

Supplementary Figure 2: DRG sensory neurons enhance MDA-MB-231 TNBC cells migration *in vitro.* a) Immunostaining of dissected and processed DRG neurons at Day 4, stained for β3-tubulin, TRPV1 and nuclei. b) Schematics for tracking the migration speed of the same cancer cells when they are on versus off neuron fiber. c) 2D migration speed of 231 cells when they are on versus off neuron fiber, each point represents the average speed of one tracked cell over 16h, n= at least 20 cells per condition. Data show mean ± SEM. Different shades of color represent cells from different biological replicates. Significance was determined by one-way ANOVA. (*$p<0.05$, ****$p<0.0001$). d) 2D migration speed of 231 cells when they are on versus off neuron fiber, with line connecting speed of the same cell. Significance was determined by paired t-test (****$p<0.0001$). e) Rose plots of 231 cell migration when cultured with conditioned media from a range of DRG sensory neurons density. f) 2D migration speed of 231 cells in DRG conditioned media. Data show mean ± SEM. Different shades of color represent cells from different biological replicates. Significance was determined by one-way ANOVA. (**$p<0.01$, ***$p<0.001$).

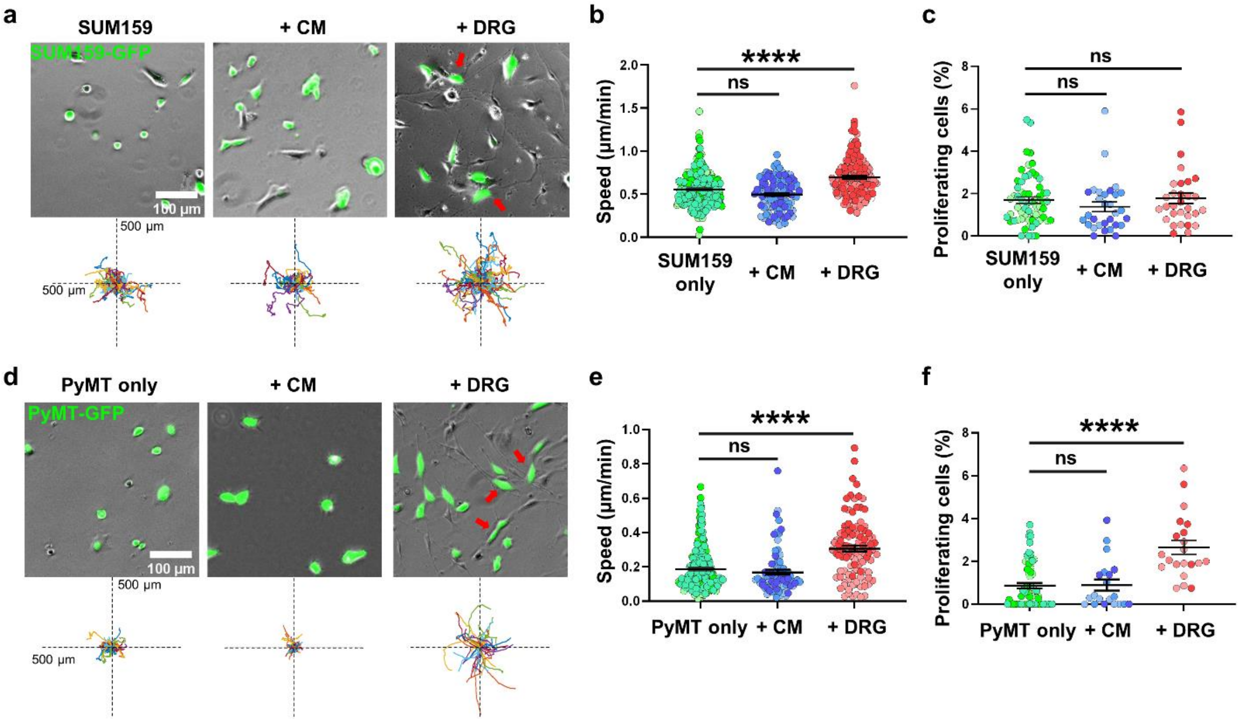

Supplementary Figure 3: DRG sensory neurons increase SUM159 and PyMT cell migration *in vitro*. a,d) Representative images and Rose plots displaying the migration track of GFP tagged SUM159 and PyMT cells over 16h. b,e) 2D migration speed of SUM159 and PyMT cells, each point represents the average speed of one tracked cell over 16h, n= at least 150 cells per condition. c,f) Quantification of SUM159 and PyMT cells undergoing proliferation in tracking period, each point represents a field of view, n = at least 15 per condition. Data show mean ± SEM. Different shades of color represent cells from different biological replicates. Significance was determined by one-way ANOVA. (****$p<0.0001$).

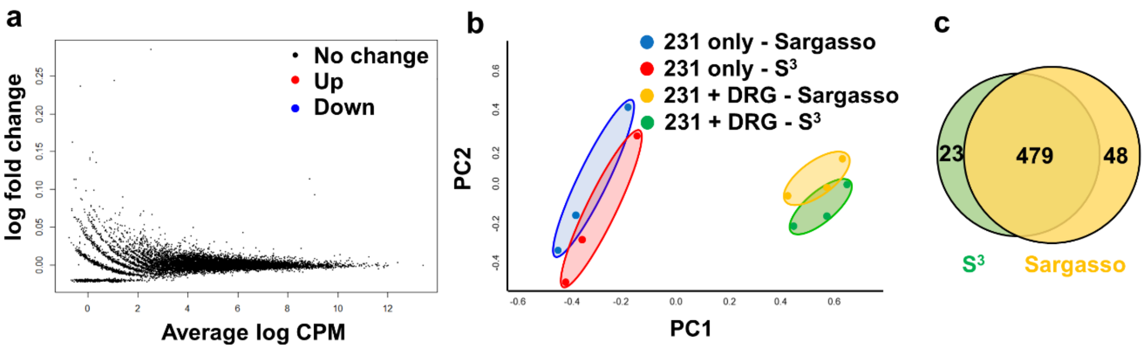

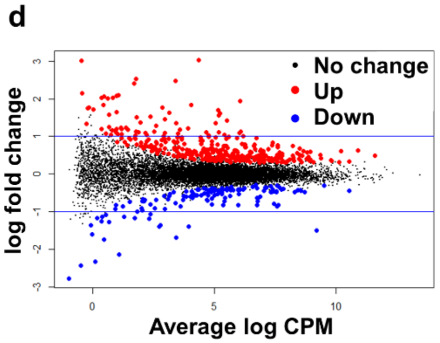

e

Cell migration		Cell adhesion	
**Genes**	**Fold change**	**Genes**	**Fold change**
FPR1	2.27	TMEM8	1.90
PLXNB1	1.92	LAMA4	1.65
COL5A1	1.62	TNC	1.50
SAA1	1.50	PLXNB3	1.48
PLXNB3	1.48	COL7A1	1.48
PTN	1.45	TGFBI	1.40
SEMA3C	1.41	LAMB1	1.33
LAMB1	1.33	COL13A1	1.32
SDC4	1.30	CLDN7	1.29
NDE1	1.25	ITGA5	1.24

Supplementary Figure 4: Robust species-specific sequencing pipelines demonstrate that MDA-MB-231 cells upregulate migration and adhesion pathways. a) Gene expression of in-silico mixed 231 cells versus 231 cells alone shows no significance difference. b) Principal component analysis of 231 only and 231 cultured with DRG gene expression processed through S^3 and Sargasso algorithm. c) Overlap of differentially expressed genes in 231 co-cultured with DRG identified by S^3 and Sargasso algorithm. d) Gene expression of 231 in co-culture versus control shows more upregulation than downregulation. e) Top 10 upregulated genes in migration and adhesion pathway in 231 cells cultured with DRG sensory neurons.

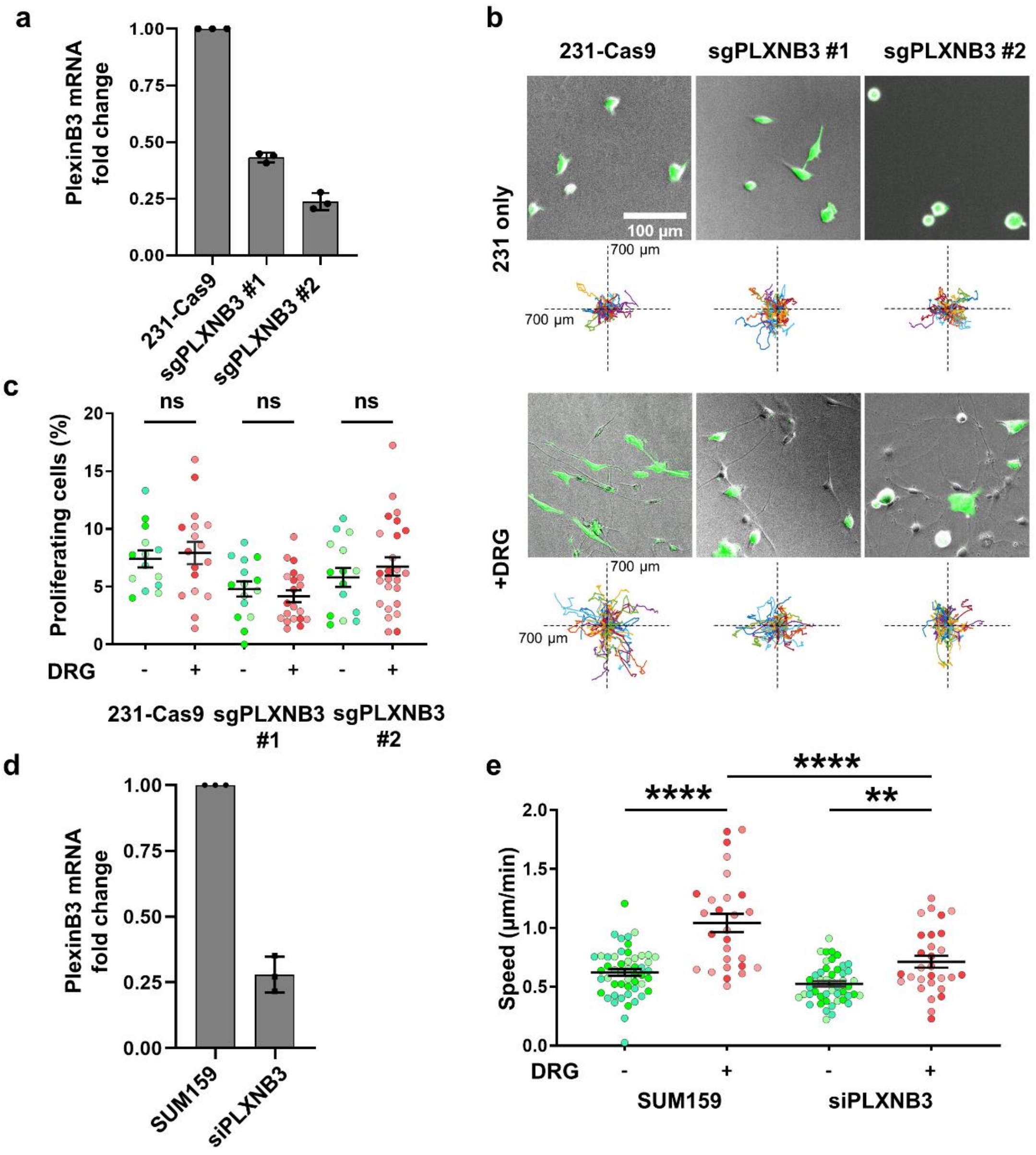

Supplementary Figure 5: Knocking down PlexinB3 inhibits DRG-induced adhesion and migration. a) Quantification of PlexinB3 expression of knocked down 231 cells by qPCR. b) Representative images and Rose plots displaying the migration track of 231-Cas9 and 231-sgPLXB3 cells over 16h. c) Quantification of 231 cells undergoing proliferation in tracking period, each point represents a field of view, n = at least 15 per condition. d) Quantification of PlexinB3 expression of knocked down SUM159 cells by qPCR. e) 2D migration speed of GFP tagged SUM159 with or without PlexinB3-targeting siRNA, each point represents the average speed of one tracked cell over 16h, n= at least 30 cells per condition. Different shades of color represent cells from different biological replicates. Data show mean ± SEM. Significance was determined by one-way ANOVA (**$p<0.01$, ****$p<0.0001$)
