# Supplementary material for: Sensory nerves enhance triple-negative breast cancer invasion and metastasis via the axon guidance molecule PlexinB3
Source: NPJ Breast Cancer. 2022 Nov 4;8:116. doi: 10.1038/s41523-022-00485-z (PMC9636220; doi:10.1038/s41523-022-00485-z)
Supplement: Supplementary file 2 — Reporting Summary [file 41523_2022_485_MOESM2_ESM.pdf]

nature portfolio

Corresponding author(s): Madeleine Oudin

Last updated by author(s): 08/05/2022

Reporting Summary

Nature Portfolio wishes to improve the reproducibility of the work that we publish. This form provides structure for consistency and transparency in reporting. For further information on Nature Portfolio policies, see our Editorial Policies and the Editorial Policy Checklist.

Statistics

For all statistical analyses, confirm that the following items are present in the figure legend, table legend, main text, or Methods section.

n/a	Confirmed	
☐	☒	The exact sample size (*n*) for each experimental group/condition, given as a discrete number and unit of measurement
☐	☒	A statement on whether measurements were taken from distinct samples or whether the same sample was measured repeatedly
☐	☒	The statistical test(s) used AND whether they are one- or two-sided<br>*Only common tests should be described solely by name; describe more complex techniques in the Methods section.*
☒	☐	A description of all covariates tested
☐	☒	A description of any assumptions or corrections, such as tests of normality and adjustment for multiple comparisons
☐	☒	A full description of the statistical parameters including central tendency (e.g. means) or other basic estimates (e.g. regression coefficient) AND variation (e.g. standard deviation) or associated estimates of uncertainty (e.g. confidence intervals)
☐	☒	For null hypothesis testing, the test statistic (e.g. *F*, *t*, *r*) with confidence intervals, effect sizes, degrees of freedom and *P* value noted<br>*Give P values as exact values whenever suitable.*
☒	☐	For Bayesian analysis, information on the choice of priors and Markov chain Monte Carlo settings
☒	☐	For hierarchical and complex designs, identification of the appropriate level for tests and full reporting of outcomes
☒	☐	Estimates of effect sizes (e.g. Cohen's *d*, Pearson's *r*), indicating how they were calculated

Our web collection on statistics for biologists contains articles on many of the points above.

Software and code

Policy information about availability of computer code

Data collection	VW-9000 Video Analysis Software: semi-automatic cell tracking
Data analysis	FastQC 0.11.9: RNSseq raw read quality control<br>STAR 2.7.3a: RNAseq read aligner<br>S^3 1.0.1: Separate RNAseq reads based on species<br>Sargasso 2.0: Separate RNAseq reads based on species<br>EdgeR 3.15: mRNA differential expression analysis<br>GSEA 4.0.3: Pathway analysis<br>GOrilla: Pathway analysis<br>Revigo: Summarize pathway analysis results<br>Custom Matlab (vR2020a) codes: calculate and visualize cell movement from XY coordinate<br>Graphpad Prism 9: Statistical analysis and visualization of data<br>ImageJ 1.53f51: Image processing and measurement

For manuscripts utilizing custom algorithms or software that are central to the research but not yet described in published literature, software must be made available to editors and reviewers. We strongly encourage code deposition in a community repository (e.g. GitHub). See the Nature Portfolio guidelines for submitting code & software for further information.

Data

Policy information about availability of data

All manuscripts must include a data availability statement. This statement should provide the following information, where applicable:

- Accession codes, unique identifiers, or web links for publicly available datasets
- A description of any restrictions on data availability
- For clinical datasets or third party data, please ensure that the statement adheres to our policy

RNA-seq data which include raw sequencing file, aligned and processed read counts, and differentially expressed genes summary are publicly available in GEO under accession code GSE180508. Patient survival data are available in kmplot.com. Other data and codes supporting the findings of this study are available from the corresponding author upon reasonable request.

Human research participants

Policy information about studies involving human research participants and Sex and Gender in Research.

Reporting on sex and gender	N/A
Population characteristics	N/A
Recruitment	N/A
Ethics oversight	N/A

Note that full information on the approval of the study protocol must also be provided in the manuscript.

Field-specific reporting

Please select the one below that is the best fit for your research. If you are not sure, read the appropriate sections before making your selection.

☒ Life sciences ☐ Behavioural & social sciences ☐ Ecological, evolutionary & environmental sciences

For a reference copy of the document with all sections, see nature.com/documents/nr-reporting-summary-flat.pdf

Life sciences study design

All studies must disclose on these points even when the disclosure is negative.

Sample size	Animal studies sample size was determined based on previous studies and power analyses. We performed a power analysis (Power and Precision 4.) and determined that 8 animals per group will be adequate for 90% power to detect a difference in burden (two–tailed alpha of 0.5).
Data exclusions	RNAseq data from one replicate of MDA-MB-231 cells in DRG conditioned media was excluded due to being a outlier. Heatmap and PCA analysis clearly identified this sample as an outlier. The full RNAseq dataset, including this outlier is available in GEO under GSE180508.
Replication	Experiments were conducted with technical and 3 biological replicates
Randomization	Mice and cells are randomly allocated
Blinding	Investigators were blinded during measurement and analysis of in vivo experiments. In vitro experiments analysis were blinded whenever possible, since DRG neurons have a clearly observable presence in co-culture.

Reporting for specific materials, systems and methods

We require information from authors about some types of materials, experimental systems and methods used in many studies. Here, indicate whether each material, system or method listed is relevant to your study. If you are not sure if a list item applies to your research, read the appropriate section before selecting a response.

Materials & experimental systems

n/a	Involved in the study
☐	☒ Antibodies
☐	☒ Eukaryotic cell lines
☒	☐ Palaeontology and archaeology
☐	☒ Animals and other organisms
☒	☐ Clinical data
☒	☐ Dual use research of concern

Methods

n/a	Involved in the study
☒	☐ ChIP-seq
☒	☐ Flow cytometry
☒	☐ MRI-based neuroimaging

Antibodies

Antibodies used

rabbit anti-beta-3-tubulin (ab18207, Abcam, Cambridge, MA)
mouse anti-beta-3-tubulin (T5758, Sigma, St. Louis, MO)
mouse anti-mouse-TRPV1 (sc-398417, Santa Cruz Biotechnology, Dallas, TX)
rabbit anti-human-TRPV1 (ab3487, Abcam, Cambridge, MA)
mouse anti-tyrosine-hydroxylase (T1299, Sigma, St.Louis, MO)
rabbit anti-GFP (A-11122, Thermo Fisher Scientific, Waltham, MA)
Alexa Flour 633 Phalloidin (A22284, Thermo Fisher Scientific, Waltham, MA)
DAPI (D1306, Thermo Fisher Scientific, Waltham, MA)

Validation

All antibodies have been validated by manufacturer with Western blot and/or immunohistochemistry.

Eukaryotic cell lines

Policy information about cell lines and Sex and Gender in Research

Cell line source(s)

MDA-MB-231 (ATCC, Mannassas, VA)
SUM159 (ATCC, Mannassas, VA)
MMTV-PyMT (Dervied from MMTV-PyMT mice model)

Authentication

MDA-MB-231 and SUM159 were authenticated by manufacter

Mycoplasma contamination

Cell lines were routinely checked for mycoplasma with Universal Mycoplasma Detection Kit every 2-3 months

Commonly misidentified lines
(See ICLAC register)

Name any commonly misidentified cell lines used in the study and provide a rationale for their use.

Animals and other research organisms

Policy information about studies involving animals; ARRIVE guidelines recommended for reporting animal research, and Sex and Gender in Research

Laboratory animals

SW-F mice, 7 weeks old
NOD-SCID-γ mice: 7 weeks old

Wild animals

N/A

Reporting on sex

Only female mice were used in this study

Field-collected samples

N/A

Ethics oversight

All animal procedures were reviewed and approved by the Tufts University Institutional Animal Care and Use Committee

Note that full information on the approval of the study protocol must also be provided in the manuscript.
